# Complete Genome Sequence of a Sub-Subgenotype 2.1i Isolate of Classical Swine Fever Virus from China

**DOI:** 10.1128/genomeA.00127-17

**Published:** 2017-04-06

**Authors:** Bikai Zhang, Shijiang Mi, Fei Bao, Huancheng Guo, Changchun Tu, Jishu Shi, Wenjie Gong

**Affiliations:** a Institute of Military Veterinary Medicine, Academy of Military Medical Sciences, Changchun, China; b College of Veterinary Medicine, Yangzhou University, Yangzhou, China; c Jiangsu Co-innovation Center for Prevention and Control of Important Animal Infectious Diseases and Zoonoses, Yangzhou, China; d Department of Anatomy and Physiology, College of Veterinary Medicine, Kansas State University, Manhattan, Kansas, USA

## Abstract

The complete genome sequence of a sub-subgenotype 2.1i isolate of classical swine fever virus (CSFV), GD317/2011, was determined. Notably, GD317/2011 is distant from the sub-subgenotype 2.1b isolate HEBZ at genes of E^rns^, E1, E2, P7, NS2, NS5A and the 3′-nontranslated region (3′-NTR) but is closely related to that at genes of N^pro^, Core, NS3, NS4A, NS4B, and NS5B.

## GENOME ANNOUNCEMENT

Classical swine fever (CSF), caused by CSF virus (CSFV), is a devastating swine infectious disease worldwide. The CSFV genome is a positive single-stranded RNA consisting of untranslated regions at both termini, the 5′-nontranslated region (5′-NTR) and 3′NTR, and a single large open reading frame (ORF) that was translated into a 3,898-amino-acid polyprotein and subsequently cleaved by viral and host proteases to generate four structural (Core, E^rns^, E1, and E2) and eight nonstructural (N^pro^, P7, NS2, NS3, NS4A, NS4B, NS5A, and NS5B) proteins (1). Based on the nucleotide sequences of partial 5′NTR and NS5B and partial or whole E2, CSFV isolates have been divided into three genotypes and 11 subgenotypes (1.1 to 1.4, 2.1 to 2.3, and 3.1 to 3.4) (2–4). In mainland China, CSFV isolates circulating in the late 1980s and 1990s consisted of four subgenotypes (1.1, 2.1, 2.2, and 2.3) (5). More recently, the genetic diversity of subgenotype 2.1 isolates was analyzed, and 10 sub-subgenotypes (2.1a to 2.1j) have been identified (6, 7). A greater understanding of the mechanisms of emergence of subgenotype 2.1 isolates will require analysis of additional complete genomes. Toward this goal, we sequenced the entire genome of sub-subgenotype 2.1i isolate GD317/2011.

GD317/2011 was isolated from a pig kidney collected from Guangdong, China, in 2011 and classified as sub-subgenotype 2.1i by phylogenetic analysis of the complete E2 gene sequences (6). To obtain the entire genome sequence of this 2.1i isolate, total RNA was extracted from a 10% homogenate of the kidney sample using the TRIzol reagent (Invitrogen, Carlsbad, CA), according to the manufacturer’s instructions. Reverse transcription, PCR amplification, and sequencing of the whole genome were conducted as previously described (8, 9). The entire genome sequence of GD317/2011 was assembled using the SeqMan program of Lasergene (DNAStar, Inc., Madison, WI). Phylogenetic analysis was performed with MEGA 6.06 (Center for Evolutionary Functional Genomes, Tempe, AZ) using the neighbor-joining method, with a bootstrap value of 1,000 repetitions.

The whole genome of GD317/2011 was found to be 12,295 nucleotides (nt) in length, consisting of a 5′-NTR of 373 nt, an ORF of 11,697 nt, and a 3′-NTR of 225 nt. Sequence comparisons indicated that GD317/2011 shared 96.0% sequence identity with sub-subgenotype 2.1b isolate HEBZ at the whole-genome level, and a comparison of their E2 gene sequences revealed greater divergence (93.3%). When individual genes of GD317/2011 were aligned with corresponding regions of other subgenotype 2.1 isolates, it was found that GD317/2011 shared 98.9%, 98.6% (98.2%), 98.3% (98.0%), 96.9% (99.1%), 96.9% (96.9%), 97.6% (99.1%) and 97.4% (98.2%) nucleotide (amino acid) sequence identity, respectively with 5′-NTR, N^pro^, Core, NS3, NS4A, NS4B, and NS5B of strain HEBZ. The similarities between GD317/2011 and HEBZ were higher than with other subgenotype 2.1 isolates. Phylogenetic analysis showed that the phylogeny of the tree constructed with the whole genome is consistent with that of E2 and that GD317/2011 clustered together with 2.1i isolate SXYL2006 in both, unlike phylogenetic trees based on the nucleotide sequences of NS4B and NS5B, in which GD317/2011 clustered with 2.1b isolate HEBZ. Therefore, the obtained complete genome sequence of GD317/2011 will be useful for further evolutionary analysis of CSFV.

### Accession number(s).

The complete genome sequence of GD317/2011 has been deposited in GenBank under the accession no. KY132096.
